# Acupuncture combined with TCM bonesetting in the treatment of distal radius fractures: A protocol for systematic review and meta-analysis

**DOI:** 10.1097/MD.0000000000028279

**Published:** 2021-12-17

**Authors:** Tongxin Liu, Yu Xia, Yitao Zhang, Gaoyan Kuang, Liangrong Zhou

**Affiliations:** a School of Humanities and Management, Hunan University of Chinese Medicine, Hunan, China; b Hunan Provincial Key Laboratory of Diagnostics in Chinese Medicine, Hunan University of Chinese Medicine, Changsha, Hunan, China; c Department of Orthopedics, The First Hospital of Hunan University of Chinese Medicine, Changsha, Hunan, China.

**Keywords:** acupuncture, bonesetting, distal radius fractures, meta-analysis, protocol, TCM, western medicine treatment

## Abstract

**Background:**

Acupuncture combined with traditional Chinese medicine (TCM) bonesetting is an effective and more acceptable treatment method for distal radius fractures; this study aims to evaluate the clinical efficacy and other relevant factors of it compared with western medicine therapy such as operation.

**Methods:**

Databases CNKI, Wanfang, VIP, SinoMed, and PubMed were searched for the current study. The retrieval time was from the establishment to November 14, 2021. Literature quality was evaluated according to the bias risk assessment criteria of Cochrane Collaboration network. RevMan 5.3 and Stata 12.0 were used to perform this research.

**Results:**

This study will appraise the effectiveness and advantages of acupuncture combined with TCM bonesetting for distal radius fractures in terms of excellent and good rate, length of stay, hospitalization expenses, complication, and other factors.

**Conclusion:**

This study provides reliable evidence-based support for the clinical efficacy and advantages of acupuncture combined with TCM bonesetting for distal radius fractures.

**OSF registration DOI:**

10.17605/OSF.IO/BUPE8.

## 1 Introduction

Distal radial fracture (DRF) is a common clinical fracture of the upper limb, most of which occurs 2 to 3 cm away from the distal radial articular surface. It is usually accompanied by swelling, tenderness, and limited movement of the wrist. With the acceleration of the aging of China's population, the incidence of this disease is also increasing year by year, which has a serious impact on the health of patients and their family.^[1]^ The current treatment methods include closed reduction, plaster or splint, surgery, etc; most patients can achieve satisfactory functional recovery.^[2]^ However, problems of high cost, poor acceptance of surgery, and more contraindications of the elderly and children remain to be solved.

From the point of view of traditional Chinese medicine (TCM), the main external cause of DRF is indirect violence, while the internal cause is mainly related to kidney deficiency and spleen deficiency.^[3]^ The method of acupuncture and bonesetting in Chinese medicine has a long history. It is one of the most important ways to treat fracture. Eight skills of bonesetting are the main treatment techniques such as touching, connecting, lifting, pressing, rubbing, and pushing.^[4]^ Acupuncture could improve the recovery of patients after surgery or with complications. This combined therapy has unique advantages in nonsurgical treatment of DRF, which is easily accepted by patients. This meta-analysis aims to further understand the curative effect and related factors of acupuncture combined with TCM bonesetting for DRF.

## 2 Methods

### 2.1 Study registration

The protocol of this systematic review and meta-analysis refers to the guidelines of the Preferred Reporting Items for Systematic Reviews and Meta-Analyses protocols (PRISMA-P).^[5]^ This protocol has been registered on OSF (registration number: DOI: 10.17605/OSF.IO/BUPE8).

### 2.2 Ethics

As the data of this review were derived from published literature, ethical approval is not required.

### 2.3 Inclusion criteria

#### 2.3.1 Types of studies

Only randomized controlled trials (RCTs) of acupuncture combined with TCM bonesetting in the treatment of DRF will be included, regardless of publication or region, but language will be restricted to Chinese and English.

#### 2.3.2 Participants

The research subjects were DRF patients, without gender, age, and race limitations.

#### 2.3.3 Interventions

The experimental groups were treated with acupuncture combined with TCM bonesetting.

#### 2.3.4 Comparison

The control groups were treated with general western medicine treatment such as surgical operation.

#### 2.3.5 Outcomes

The primary outcomes included excellent and good rate, total effective rate, and TCM syndrome score. The TCM syndrome score scale is composed of 23 items to evaluate. Each item is worth 1 to 4 points, so the total TCM syndrome scores that each patient earned ranged from 23 (best) to 72 (worst).

The secondary outcomes included length of stay, hospitalization expenses, and complication.

### 2.4 Exclusion criteria

Exclusion criteria included:

(1) Repeated publications.
(2) The data are incomplete and cannot be extracted for analysis.
(3) Unclear outcome.
(4) Animal experiments, cell experiments, and the review literature.
(5) Case reports.
(6) The control group was combined with other TCM treatments.

### 2.5 Information sources and literature search

On the basis of the Preferred Reporting Items for Systematic Reviews and Meta-Analyses guidelines, the meta-analysis is performed. Databases searched included Chinese databases CNKI, Wanfang, VIP and English databases PubMed, Embase and Cochrane Library. The retrieval time is from the establishment to November 2021. Chinese search terms: “distal radius fractures (rao gu yuan duan gu zhe)” AND “traditional Chinese medicine bonesetting (zhong yi zheng gu)” AND “acupuncture (zhen jiu)” AND “clinical trial (lin chuang guan cha)” OR “randomized controlled trial (sui ji dui zhao shi yan)”; English search terms: “distal radius fractures” AND “traditional Chinese medicine bonesetting” OR “TCM bonesetting” AND “acupuncture” AND “clinical trial” OR “randomized controlled trial”. Taking PubMed as an example, the search strategy is listed in Table 1.

**Table 1:** Search strategy for PubMed database.

Number	Search items
#1	distal radius fracture
#2	acupuncture
#3	traditional Chinese medicine bonesetting
#4	TCM bonesetting
#5	#3 OR #4
#6	clinical trial
#7	randomized controlled trial
#8	#6 OR #7
#9	#1 AND #2 AND #5 AND #8

### 2.6 Literature selection

**Figure 1:**
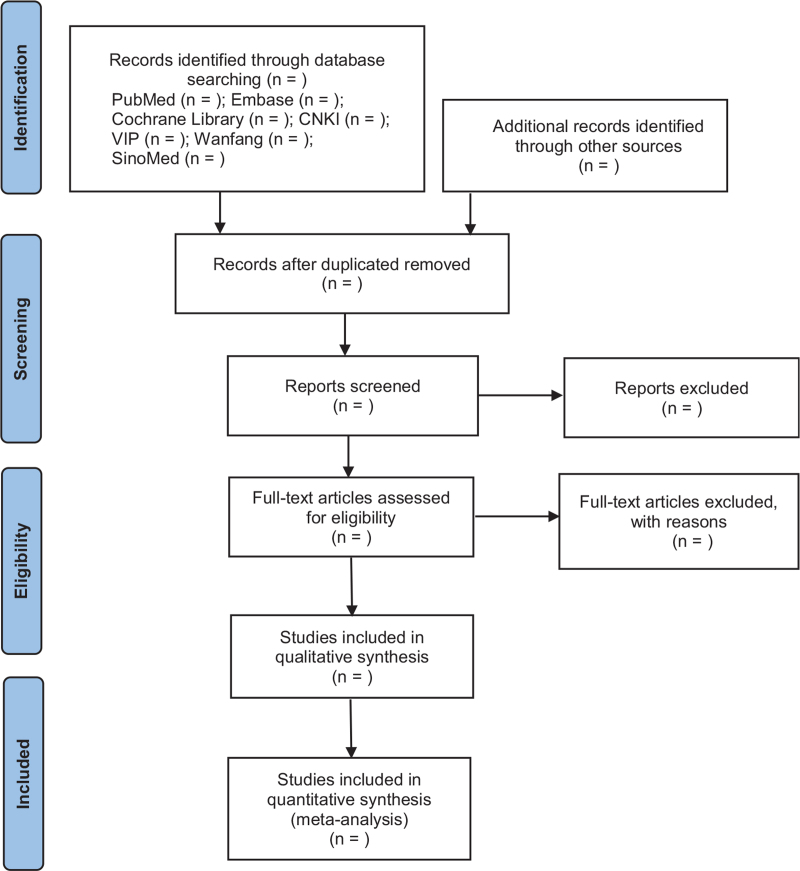
Flow chart showing literature screening process.

Data extraction and quality assessment are conducted by 2 researchers independently according to the screening criteria, then cross-checked the data. If there are conflicts of opinions, resolve them through collective discussion. In the process of literature screening, the literature with irrelevant titles is excluded, and the abstracts and full texts should be further read to determine the final included literature. The process of literature screening is shown in Figure 1.

### 2.7 Data extraction and quality assessment

The basic information contained: first author, year of publication, sample size, interventions, and course of treatment. Bias risk assessment included random grouping methods, allocation concealment, blinding methods, incomplete data, and each is rated as “high risk,”, “low risk,” or “unclear.” Literature quality is evaluated according to the bias risk assessment criteria of Cochrane Collaboration network.

### 2.8 Statistical analysis

RevMan 5.3 and Stata 12.0 are used for this meta-analysis. The calculation of the dichotomous variable is expressed by odds ratio (OR), and mean difference (MD) or standardized mean difference (SMD) is calculated for the continuous variable. 95% confidence interval (95% CI) is expressed for the numerical value. Heterogeneity is evaluated by *I*^*2*^ and chi-square tests. The fixed-effects model is used for analysis if *I*^*2*^ ≤ 50%; the random-effects model is used if *I*^*2*^ > 50%. The results are shown in the forest plot. For all outcomes, publication bias is assessed by Egger and Begg tests.

### 2.9 Sensitivity evaluation

The sensitivity of the meta-analysis is evaluated by changing the effect model, and the changed OR and MD (SMD) values are used for the sensitivity analysis.

### 2.10 Grading the quality of evidence

The Grading of Recommendation, Assessment, Development, and Evaluation (GRADE) system^[6]^ will be used to appraise the quality of evidence from the researches obtained. The levels of it will be divided into high, moderate, low, very low.

## 3 Discussion

DRF is an common disease in elderly people, with an incidence of 200 to 1200 per 100,000 person each years.^[7,8]^ DRF is the second most common fractures after hip fractures in patients over 65 years of age, accounting for almost one-fifth of all fractures in this age group.^[9]^

The purpose of this treatment of DRF is to recover function as close as possible to the level before the fracture. At present, there are many treatments for DRF. However, it can be broadly divided into 2 categories: conservative treatment and surgical treatment. Traditionally, nonsurgical treatment has involved reduction of the fracture near the anatomic site followed by fixation with a functional cast for 4 to 5 weeks.^[10]^ Although there are more surgical treatment than nonsurgical treatment, for DRF, conservative treatment should be considered first.^[11]^ There was no significant statistical difference between conservative treatment and surgical treatment in the overall evaluation of later stage, and conservative treatment could avoid the related complications caused by surgery.^[12]^ Acupuncture and TCM bonesetting therapy has a history of more than 2000 years and is one of the most important treasures in the development of Chinese medicine. Patients with DRF receive the treatment of acupuncture and TCM bonesetting, which can achieve better therapeutic effect and help the reduction of fracture position. Acupuncture plays the role of activating the tendons and vessels, promoting the growth rate of bone and the healing speed; on this basis, the selection of the best TCM bonesetting method can greatly reduce the occurrence of complications and reduce the pain of patients.^[13,14]^

However, there are some limitations of this study. The limited sample size may influence the comprehensiveness of the whole results. In addition, slight publication bias and the low quality of RCTs of the included studies may diminish the power of this meta-analysis. Therefore, more well-designed, rigorously conducted and RCTs of high quality are needed to verify the efficacy and advantages of acupuncture combined with TCM bonesetting as a good treatment of DRF.

## Author contributions

**Conceptualization:** Tongxin Liu.

**Data curation:** Tongxin Liu and Yitao Zhang, Yu Xia.

**Data curation:** Tongxin Liu, Yitao Zhang.

**Funding acquisition:** Liangrong Zhou.

**Investigation:** Yitao Zhang, Gaoyan Kuang.

**Literature retrieval:** Tongxin Liu and Yu Xia.

**Software:** Tongxin Liu and Yu Xia.

**Supervision:** Liangrong Zhou.

**Writing – original draft:** Tongxin Liu.

**Writing – review & editing:** Liangrong Zhou.
